# SIRT1 Regulates HIV Transcription via Tat Deacetylation

**DOI:** 10.1371/journal.pbio.0030041

**Published:** 2005-02-08

**Authors:** Sara Pagans, Angelika Pedal, Brian J North, Katrin Kaehlcke, Brett L Marshall, Alexander Dorr, Claudia Hetzer-Egger, Peter Henklein, Roy Frye, Michael W McBurney, Henning Hruby, Manfred Jung, Eric Verdin, Melanie Ott

**Affiliations:** **1**Gladstone Institute of Virology and Immunology, University of CaliforniaSan Francisco, CaliforniaUnited States of America; **2**Applied Tumorvirology, Deutsches KrebsforschungszentrumHeidelbergGermany; **3**Institute of Biochemistry, Humboldt UniversityBerlinGermany; **4**Department of Pathology, University of PittsburghPittsburgh, PennsylvaniaUnited States of America; **5**Ottawa Regional Cancer CentreOttawaCanada; **6**Department of Pharmaceutical Sciences, Albert-Ludwigs-UniversityFreiburgGermany; Fred Hutchinson Cancer Research CenterUnited States of America

## Abstract

The human immunodeficiency virus (HIV) Tat protein is acetylated by the transcriptional coactivator p300, a necessary step in Tat-mediated transactivation. We report here that Tat is deacetylated by human sirtuin 1 (SIRT1), a nicotinamide adenine dinucleotide-dependent class III protein deacetylase in vitro and in vivo. Tat and SIRT1 coimmunoprecipitate and synergistically activate the HIV promoter. Conversely, knockdown of SIRT1 via small interfering RNAs or treatment with a novel small molecule inhibitor of the SIRT1 deacetylase activity inhibit Tat-mediated transactivation of the HIV long terminal repeat. Tat transactivation is defective in SIRT1-null mouse embryonic fibroblasts and can be rescued by expression of SIRT1. These results support a model in which cycles of Tat acetylation and deacetylation regulate HIV transcription. SIRT1 recycles Tat to its unacetylated form and acts as a transcriptional coactivator during Tat transactivation.

## Abstract 2

Cycles of Tat acetylation and deacetylation, mediated by human sirtuin 1 (SIRT1), regulate HIV transcription suggesting that SIRT1 could be a therapeutic target

## Introduction

The Tat protein of human immunodeficiency virus 1 (HIV-1) is essential for the transcriptional activation of the integrated HIV-1 provirus. Without Tat, HIV transcriptional elongation is inefficient and results in abortive transcripts that cannot support viral replication [1,2]. Tat is produced early after infection from rare full-length genomic transcripts generated despite the elongation defect. These transcripts lead to the synthesis of a few Tat molecules sufficient to stimulate HIV transcription elongation, leading to the production of additional Tat transcripts and protein.

Tat activates HIV transcription through the *trans*-acting responsive element (TAR), an RNA stem-loop structure that forms at the 5′ end of all viral transcripts [3,4]. The TAR stem contains a three-nucleotide bulge structure recognized by the arginine-rich motif (ARM) in Tat (amino acids 49–57). In vivo, Tat binding to TAR requires cyclinT1, a cofactor that interacts cooperatively with both the N-terminal transactivation region of Tat (amino acid 1–48) and loop sequences at the top of the TAR stem-loop structure [5]. CyclinT1, a component of pTEFb (the positive transcription elongation factor b), recruits the cyclin-dependent kinase 9 (CDK9) to the HIV promoter. CDK9 hyperphosphorylates the C-terminal domain of RNA polymerase II, which potently enhances the processivity of the RNA polymerase II complex [6].

We and others have shown that Tat is acetylated at lysine 50 by the transcriptional coactivators p300 and human GCN5 (general control of amino acid synthesis 5) [7,8,9,10]. Tat acetylation is important for Tat activity and defines a critical cyclinT1-independent step in Tat transactivation [11]. Tat acetylated at lysine 50 cannot form a ternary complex with cyclinT1 and TAR RNA. It dissociates from TAR and binds instead to the p300/CREB-binding protein-associated factor (PCAF) via its bromodomain [12,13].

Our current working model is that Tat acetylation disrupts the Tat/TAR/cyclinT1 complex and leads to the transfer of Tat and PCAF to the elongating polymerase. According to this model, both forms of Tat, unacetylated and acetylated, play distinct roles in the HIV promoter transcriptional cycle and lead to the sequential recruitment of the cofactors cyclinT1 and PCAF. Because of the limiting amounts of Tat protein in the early stages of HIV infection and the critical role of unacetylated Tat for pTEFb recruitment to TAR, the question arises whether Tat acetylation can be reverted via a cellular Tat deacetylase.

There are three distinct classes of human histone deacetylases (HDACs) based on their homology with yeast transcriptional repressors. Class I and II HDACs are homologous to the yeast proteins Rpd3p (reduced potassium dependency 3) and Hda1p (histone deacetylase A1), respectively [14,15]. The deacetylase activity of class I and II HDACs is efficiently inhibited by trichostatin A (TSA) and other related hydroxamate-based inhibitors.

Class III HDACs, also named sirtuins (SIRTs), are homologous to the yeast transcriptional repressor silent information regulator 2p (Sir2p) [16]. Sir2p is a TSA-insensitive histone deacetylase that requires nicotinamide adenine dinucleotide (NAD^+^) as a cofactor [17,18,19]. Seven homologs of Sir2p have been identified in the human genome. Called SIRT1–7, they all contain a highly conserved catalytic domain [20]. Despite their enzymatic activity on histone substrates in vitro, recent experimental evidence suggests that SIRT proteins predominantly target nonhistone proteins for deacetylation, in both the nucleus and the cytoplasm. The nuclear SIRT1 protein deacetylates p53 [21,22,23], TAF_I_68 (Tata box-binding protein-associated factor I of 68 kDa) [24], PCAF and myoblast determination protein (MyoD) [25], p300 [26] and Forkhead transcription factors [26,27], the p65 subunit of nuclear factor kappa B (NF-κB) [28], and the Ku70 telomeric protein (also known as the thyroid autoantigen of 70 kDa or Ku antigen) [29]. The cytoplasmic SIRT2 protein is found associated with the microtubule network and deacetylates lysine 40 of α-tubulin [30]. SIRT3 is a mitochondrial matrix protein whose target has not been identified [31,32].

Here, we identify the class III HDAC SIRT1 as a specific Tat deacetylase and demonstrate that SIRT1 is a novel cofactor necessary for efficient Tat-mediated transactivation of the HIV promoter.

## Results

**Figure 1:**
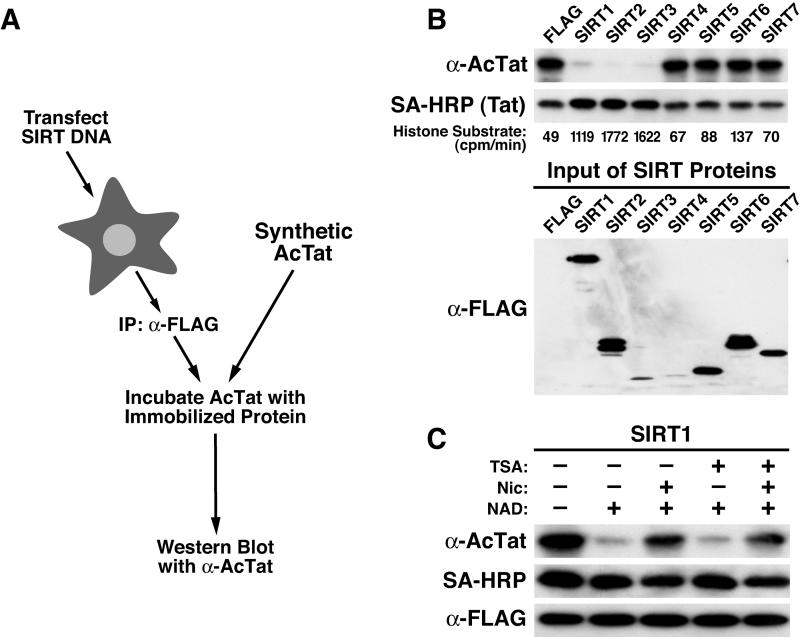
In Vitro Tat Deacetylation by Human SIRT Proteins (A) Scheme of Tat deacetylation assay with immunoprecipitated SIRT1–7 proteins. Expression vectors for FLAG-tagged SIRT proteins were transfected into HEK 293 cells, immunoprecipitated, and incubated with synthetic Tat (72 amino acids) carrying an N-terminal biotin label and an acetyl group at position 50 (AcTat) in the presence of NAD^+^. Immunoprecipitated material was also analyzed in a radioactive (^3^H) histone deacetylase assay using an H3 peptide as a substrate. (B) WB analysis of deacetylation reactions with antibodies specific for acetylated lysine 50 in Tat (α-AcTat), with SA-HRP, or with α-FLAG antibodies. (C) WB of Tat deacetylation by immunoprecipitated SIRT1 in the presence or absence of NAD^+^, TSA, or nicotinamide (Nic).

To test the ability of SIRT1–7 to deacetylate Tat in vitro, we transfected HEK 293 cells with expression vectors for human SIRT1–7 and immunoprecipitated the FLAG-tagged proteins (Figure 1A). The immunoprecipitated material was incubated with a full-length synthetic Tat protein carrying an acetylated lysine at position 50 (AcTat). The extent of Tat deacetylation was determined by Western blot (WB) with antibodies specific for the acetylated ARM in Tat [11]. Incubation of AcTat with immunoprecipitated SIRT1, SIRT2, and SIRT3 resulted in deacetylation of Tat lysine 50 (Figure 1B). These enzymes also deacetylate histones as determined in a standard histone deacetylase assay (Figure 1B). All reactions contained equal amounts of AcTat as determined by immunoblotting with streptavidin-horseradish peroxidase conjugate (SA-HRP), which recognized the biotin label attached to the N terminus of AcTat (SA-HRP in Figure 1B). SIRT enzymes in the reactions were visualized by immunoblotting with FLAG antibodies (FLAG in Figure 1B).

SIRT2 and SIRT3 proteins are localized primarily in the cytoplasm and the mitochondria [30,31], and SIRT1 resides in the cell nucleus [23,33]. Since Tat is a predominantly nuclear protein, we focused our efforts on SIRT1. The SIRT1-mediated deacetylation of Tat was dependent on NAD^+^ and completely inhibited by nicotinamide, an inhibitor for class III HDACs [34,35]. TSA, a specific inhibitor of class I and II HDACs, had no effect (Figure 1C). These results demonstrate that the Tat deacetylase activity within immunoprecipitated SIRT1 material can be solely attributed to SIRT1 and not to a contaminating class I or II HDAC.

**Figure 2:**
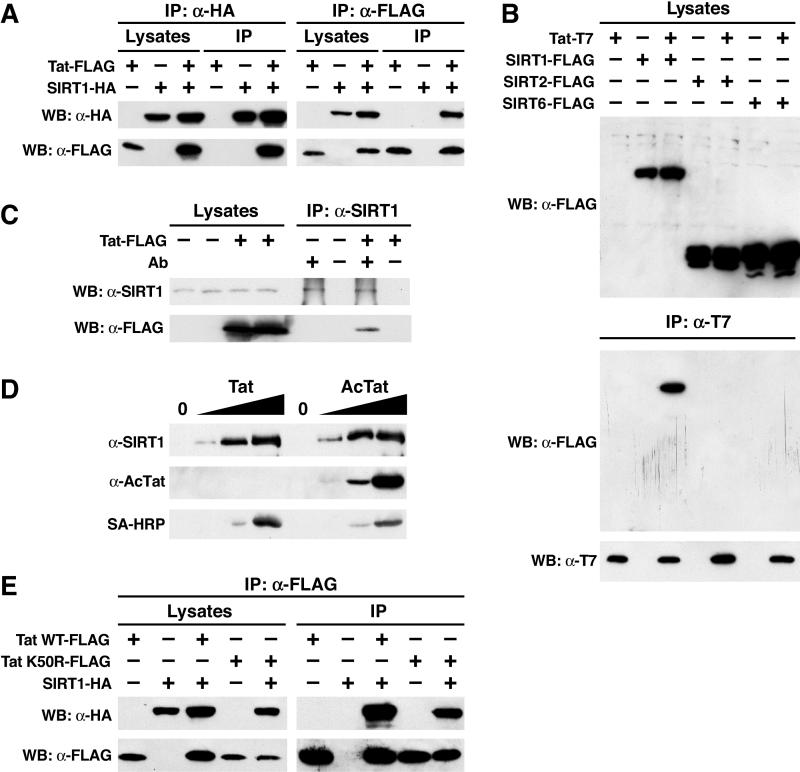
Physical Interaction between Tat and SIRT1 (A) Immunoprecipitation (IP) and WB of FLAG-tagged Tat (Tat-FLAG) and HA-tagged SIRT1 (SIRT1-HA) after transfection of corresponding expression vectors (+) or empty vector controls (−) into HEK 293 cells. (B) The same experiments as in (A) performed with T7-tagged Tat and FLAG-tagged SIRT1, SIRT2, and SIRT6. (C) Coimmunoprecipitation of FLAG-tagged Tat with endogenous SIRT1 in HEK 293 cells transfected with the Tat expression vector or the empty vector control. IPs were performed with or without rabbit α-SIRT1 antibodies. (D) WB of recombinant SIRT1 protein after pulldown with synthetic biotinylated Tat or AcTat. Tat proteins were detected with antibodies specific for acetylated lysine 50 in the Tat ARM (α-AcTat) or SA-HRP. (E) Immunoprecipitation/WB of FLAG-tagged Tat or TatK50R and HA-tagged SIRT1. WT, wild type.

To test whether Tat and SIRT1 interact, Tat/FLAG and SIRT1/influenza hemagglutinin (HA) were overexpressed in HEK 293 cells, and cellular lysates subjected to coimmunoprecipitation assays. Tat was detected with an α-FLAG antiserum in material immunoprecipitated with SIRT1 by the α-HA antibody in cells transfected with SIRT1- and Tat expression vectors, but no signal was obtained when SIRT1 or Tat alone was expressed (IP: α-HA in Figure 2A). Conversely, SIRT1 also specifically coimmunoprecipitated with Tat/FLAG (IP: α-FLAG in Figure 2A). The same was observed when Tat/T7 was coexpressed with SIRT1/FLAG and was immunoprecipitated with α-T7 antibodies (Figure 2B). No coimmunoprecipitation of Tat was observed with SIRT2 and SIRT6 (Figure 2B), two SIRT proteins that can also localize to the cell nucleus (BN and EV, personal communication), or any other SIRT protein (unpublished data). Furthermore, Tat coimmunoprecipitated with endogenous SIRT1 in Tat-expressing, but not in vector-transfected, HEK 293 cells (Figure 2C). No SIRT1- or Tat-specific signals were obtained after immunoprecipitations (IPs) in the absence of α-SIRT1 antibodies, excluding nonspecific binding of Tat to the Sepharose beads to which the antibodies were bound.

To test whether Tat and SIRT1 interact directly, increasing amounts of biotinylated synthetic Tat (72 amino acids) were incubated with recombinant full-length SIRT1. After pulldown with streptavidin-conjugated agarose, SIRT1 coimmunoprecipitated with Tat in a dose-dependent manner (Figure 2D). Recombinant SIRT1 bound equally well to acetylated and unacetylated synthetic Tat, indicating that the interaction occurred independently of the acetylation state of Tat (Figure 2D). WB with AcTat antibodies showed that AcTat remained acetylated during incubation with the SIRT1 enzyme (Figure 2D). Re-blotting with SA-HRP detected both Tat proteins in equivalent amounts in the binding reactions (Figure 2D). We also tested the ability of a Tat mutant protein (termed TatK50R to indicate mutation of lysine to arginine at position 50 of the Tat protein) to interact with SIRT1. This mutation preserves the basic charge at position 50, but cannot be acetylated. After transfection into HEK 293 cells, TatK50R accumulated to lower concentrations than wild-type Tat, but was bound to SIRT1 efficiently in coimmunoprecipitation assays (Figure 2E). These results collectively indicate that Tat binds SIRT1 directly and independently of lysine 50.

**Figure 3:**
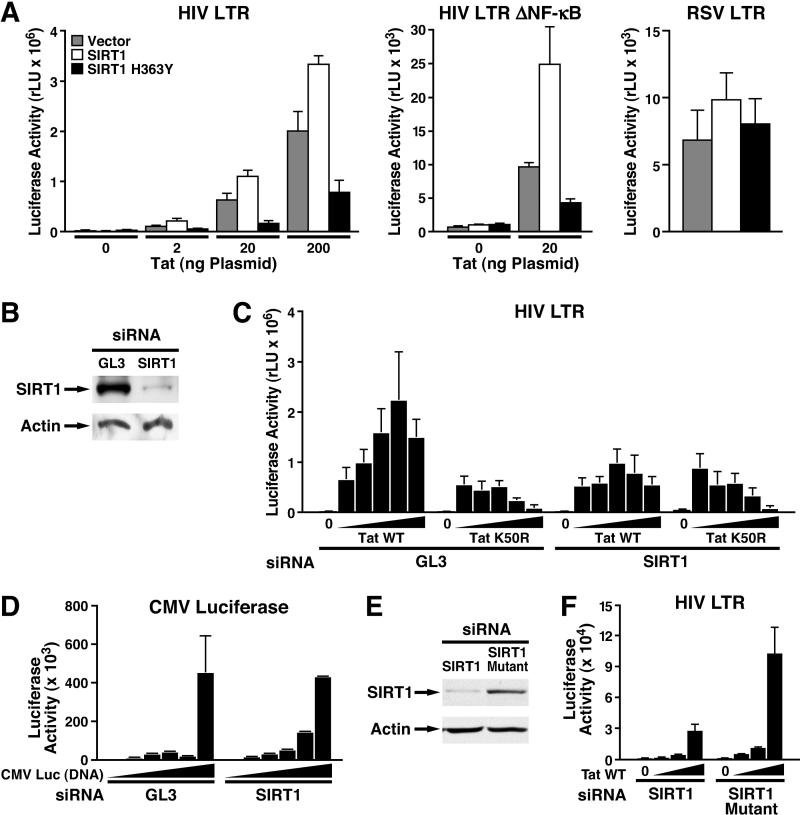
SIRT1 Is a Positive Cofactor for Tat Transactivation (A) Cotransfection of SIRT1 or the catalytically inactive SIRT1 mutant SIRT1H363Y with the HIV LTR luciferase construct and increasing amounts of a Tat expression vector (RSV-Tat: 0, 2, 20, and 200 ng), an HIV LTR luciferase construct containing mutated binding sites for the transcription factor NF-κB and RSV-Tat (20 ng), or with an RSV-luciferase construct (200 ng) in HeLa cells. The average of three experiments is shown (± standard error of the mean [SEM]). (B) WB analysis of HeLa cells 72 h after transfection of siRNAs directed against SIRT1 or GL3 control siRNAs. (C) Cotransfection of the HIV LTR luciferase construct with increasing amounts of CMV-Tat or CMV-TatK50R (0, 50, 100, 200, 400, and 800 ng) 48 h after transfection of double-stranded siRNAs directed against SIRT1 or GL3 control siRNAs in HeLa cells. Luciferase activity was measured 24 h after plasmid transfection and 72 h after siRNA transfection. Note that all luciferase reporter vectors used in this study are based on the pGL2 luciferase vector, which is not affected by GL3-specific siRNAs [36]. The average of three experiments is shown (± SEM). (D) The transcriptional activity of increasing amounts of the CMV-luciferase reporter (0, 50, 100, 200, 400, and 800 ng) was similar in SIRT1 knockdown or GL3-treated control cells. The average of two experiments performed in duplicate is shown (± SEM). (E) WB of endogenous SIRT1 or actin 72 h after transfection of siRNA directed against SIRT1 or mutated SIRT1 siRNA. (F) Cotransfection of the HIV LTR luciferase with increasing amounts of CMV-Tat (0, 2, 20, and 200 ng) in HeLa cells pretransfected with wild-type or mutant SIRT1 siRNA oligonucleotides as described in (C). WT, wild-type.

The effects of SIRT1 on Tat function were assessed after transfection into HeLa cells. SIRT1 modestly, but reproducibly, enhanced Tat-mediated transactivation of an HIV promoter luciferase construct (HIV LTR in Figure 3A). In contrast, expression of a catalytically inactive SIRT1 protein (termed SIRT1H363Y to indicate mutation of histidine to tyrosine at position 363 of the SIRT1 protein) suppressed Tat transactivation in a dominant-negative manner, indicating that the catalytic activity of SIRT1 is necessary for Tat transactivation. Similar results were obtained when an HIV promoter reporter construct containing mutant binding sites for the transcription factor NF-κB was used (HIV LTR ΔNF-κB in Figure 3A). This result indicates that the superinduction of Tat activity by wild-type SIRT1 and the suppression of Tat activity by catalytically inactive SIRT1 were dependent on the interaction between SIRT1 and Tat rather than on the interaction between SIRT1 and NF-κB/p65 [28]. Importantly, SIRT1 (both wild-type and SIRT1H363Y mutant) had no effect on the transcriptional activity of the Rous sarcoma virus (RSV) LTR, a promoter used to drive Tat expression in these cotransfection experiments.

The effect of SIRT1 on Tat transactivation was further examined using small interfering RNA (siRNA)-mediated knockdown of SIRT1. HeLa cells were transfected with double-stranded RNA oligonucleotides directed against SIRT1 or against firefly luciferase expressed from the pGL3 vector as a control. All luciferase reporter constructs described in this study are based on the pGL2 vector, which is not affected by GL3 siRNAs (siRNAs directed against firefly luciferase expressed from the pGL3 vector) [36]. Levels of endogenous SIRT1 were markedly reduced at 72 h after transfection of siRNAs specific for SIRT1 (Figure 3B). At that time, a significant decrease in Tat transactivation was noted in cells that had received the SIRT1 siRNA, but not the GL3 siRNA (Figure 3C). The SIRT1 siRNA slightly enhanced the basal HIV promoter activity without Tat, and had no effect on the transcriptional activity of TatK50R, the Tat mutant that cannot be acetylated (Figure 3C). Loss of SIRT1 had no effect on the transcriptional activity of the immediate early promoter of the cytomegalovirus (CMV) used to drive Tat expression in these experiments (Figure 3D). In addition, Tat levels in HeLa cells transfected with SIRT1 siRNAs were comparable to Tat levels detected in cells transfected with GL3 siRNAs as determined by WB (unpublished data). To confirm the specificity of the SIRT1 siRNA, mutant double-stranded siRNA oligonucleotides were generated which contained a two-nucleotide mismatch between the target mRNA for SIRT1 and the antisense strand of the siRNA. Transfection of mutant SIRT1 siRNA did not affect expression of endogenous SIRT1 protein in HeLa cells, indicating that the mutation abrogated SIRT1 mRNA cleavage (Figure 3E). SIRT1 siRNA, but not mutant siRNA, suppressed Tat transactivation of the HIV LTR luciferase construct, confirming that the observed suppression was dependent on the loss of SIRT1 protein (Figure 3F).

**Figure 4:**
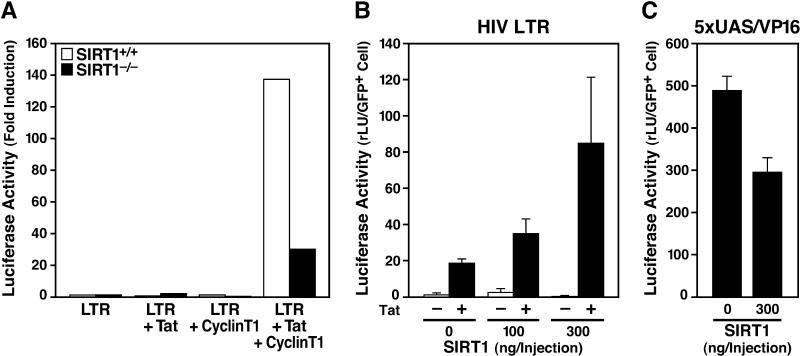
Impaired Tat Transcriptional Activity in Murine SIRT1^−/−^ Cells (A) Nuclear microinjection of HIV LTR luciferase, RSV-Tat, and a human cyclinT1-expressing construct into MEFs derived from SIRT^+/+^ or SIRT^−/−^ mice. In all experiments, a fixed amount of DNA was injected by adding the empty vector control. Cells were coinjected with CMV-GFP, and the luciferase activity per GFP-positive cell was calculated. An average of two injections is shown. (B) The HIV LTR luciferase construct together with RSV-Tat and the cyclinT1-expressing construct were coinjected into SIRT^−/−^ MEFs in the presence of increasing amounts of a plasmid expressing human SIRT1. The average of three experiments is shown (± SEM). (C) Coinjection of the human SIRT1 expression vector together with the 5xUAS luciferase construct containing five Gal4 binding sites upstream of the thymidine kinase promoter and a Gal4-VP16 expression plasmid into SIRT1^−/−^ MEFs. The average of three experiments is shown (± SEM).

Since SIRT1 only modestly enhanced Tat transactivation in HeLa cells, which already express significant levels of SIRT1, we examined the effect of SIRT1 on Tat transactivation in a SIRT1-negative background. We obtained mouse embryonic fibroblasts (MEFs) derived from SIRT1 knockout mice [37]. The HIV LTR luciferase reporter and the Tat expression vector were introduced into these cells by nuclear microinjections because of their low responsiveness to various transfection protocols. Because murine cyclinT1 does not support Tat transactivation [38,39], an expression vector for human cyclinT1 was included in the microinjections. A 120-fold increase in HIV promoter luciferase activity was detected in the presence of Tat and human cyclinT1 in SIRT1^+/+^ MEFs (Figure 4A). In contrast, Tat-mediated transactivation of the HIV LTR was reduced in SIRT1^−/−^ MEFs (Figure 4A). Ectopic expression of increasing amounts of human SIRT1 resulted in a dose-dependent increase of Tat transactivation in SIRT1^−/−^ MEFs (Figure 4B). In contrast, transactivation of the 5xUAS promoter by Gal4-VP16 was reduced in response to SIRT1 (Gal4-VP16 is a fusion between the binding domain of the DNA-binding transcription factor required for the activation of the *GAL* genes in response to galactose in Saccharomyces cerevisiae [termed Gal4], and the activator domain of the herpes simplex virus transactivator protein [designated VP16]) (Figure 4C). These results collectively demonstrate that SIRT1 represents a positive factor for Tat function.

**Figure 5:**
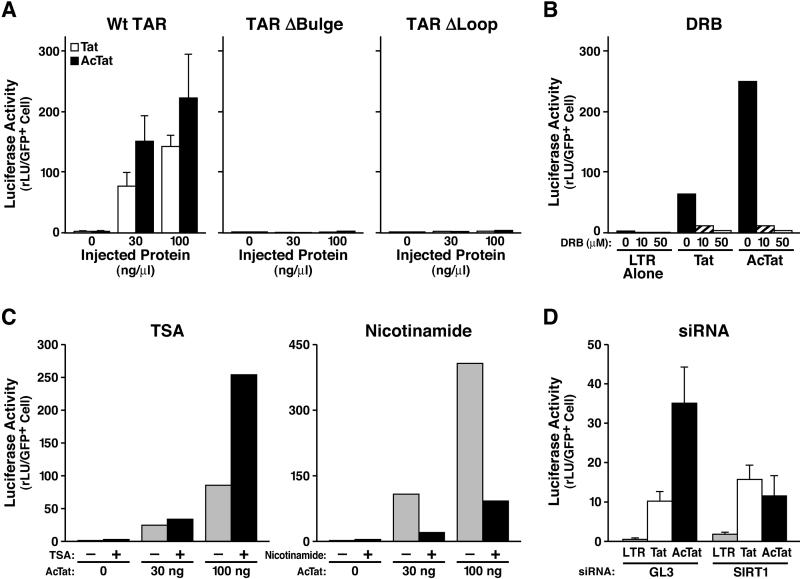
Transcriptional Activity of AcTat Depends on Deacetylation by SIRT1 (A) AcTat functions through TAR and cyclinT1 binding. Nuclear microinjection of increasing amounts of synthetic Tat or AcTat together with wild-type (wt TAR), TAR Δbulge, or TAR Δloop mutant HIV LTR luciferase constructs into HeLa cells. Cells were coinjected with CMV-GFP, and luciferase activity was calculated per GFP-positive cell. An average of three experiments is shown (± SEM). (B) AcTat transactivation requires CDK9. HeLa cells microinjected with Tat or AcTat (each 30 ng/μl) and the HIV LTR luciferase reporter were treated with increasing amounts of DRB, a known CDK9 inhibitor, for 4 h. (C) AcTat transcriptional activity is inhibited by nicotinamide, but not TSA. HeLa cells injected with HIV LTR luciferase and increasing amounts of AcTat were treated with TSA (400 nM) or nicotinamide (5 mM) for 4 h. The average of two experiments is shown. (D) SIRT1 is necessary for AcTat, but not Tat function. HeLa cells were transfected with siRNAs specific for SIRT1 or GL3 control siRNAs 48 h before microinjection of HIV LTR luciferase and Tat or AcTat (each 30 ng/μl). The average of three experiments is shown (± SEM).

This model was further tested in nuclear microinjection experiments using synthetic full-length Tat and AcTat. Microinjection of increasing amounts of either Tat or AcTat proteins into HeLa cells caused a marked transactivation of the HIV LTR luciferase reporter in a dose-dependent manner (Wt TAR in Figure 5A). AcTat transactivated the HIV promoter approximately 1.5–3-fold better than Tat. Transactivation by Tat and AcTat was dependent on the bulge and loop regions of TAR, indicating that transactivation by both proteins required the formation of an intact Tat/TAR/cyclinT1 complex [4,5,40] (TAR ΔBulge and TAR ΔLoop in Figure 5A). In agreement with this conclusion, transactivation by both Tat proteins was inhibited in a dose-dependent manner by 5,6-dichlorobenzimidazole riboside (DRB), a CDK9 inhibitor known to block Tat function (Figure 5B) [6].

According to our working model, AcTat represents a second step in the transactivation cycle [11]. Since AcTat cannot form the trimolecular complex with cyclinT1 and TAR RNA in vitro, we hypothesized that AcTat becomes partially deacetylated by the Tat deacetylase upon microinjection. This would allow the initiation of the transactivation process by unacetylated Tat binding to TAR with cyclinT1 and CDK9. To further test this hypothesis, we treated cells with deacetylase inhibitors after microinjection of AcTat and the HIV promoter construct. Treatment with TSA, an inhibitor of class I and II HDACs, enhanced the transcriptional activity of AcTat as well as the basal HIV promoter activity (TSA in Figure 5C). In contrast, nicotinamide, an inhibitor of class III deacetylases, blocked transactivation of the HIV promoter by AcTat while stimulating basal HIV promoter activity (Nicotinamide in Figure 5C). Similarly, knockdown of SIRT1 using siRNA inhibited transcriptional activity of AcTat, while slightly enhancing Tat-mediated or basal transcriptional activity of the HIV promoter (Figure 5D). These results support the model that the transcriptional activity of AcTat depends on deacetylation by SIRT1 in cells.

**Figure 6:**
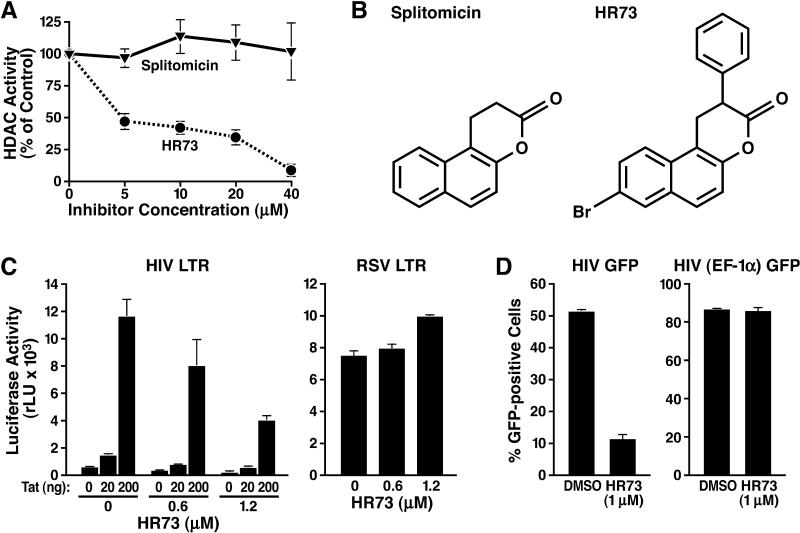
Inhibition of HIV Gene Expression by a Small Molecule Inhibitor of SIRT1 (A) In vitro histone deacetylation assays including recombinant SIRT1, radioactively labeled histone H3 peptide, and indicated concentrations of splitomicin or HR73. The average (± SEM) of two experiments performed in duplicate is shown for each point. (B) Chemical structures of splitomicin and its derivative HR73. (C) Inhibition of Tat transactivation by HR73. RSV-Tat (0, 20, and 200 ng) and HIV LTR luciferase (200 ng) or RSV-luciferase (200 ng) vectors were transfected into HeLa cells. Transfected cells were treated with indicated concentrations of HR73 or DMSO for 8 h. (D) Inhibition of HIV gene expression by HR73. GFP expression in Jurkat T cells infected with HIV_NL4–3_ containing the GFP open reading frame in place of the viral *nef* gene or with an HIV-based lentiviral vector expressing GFP from the heterologous EF-1α promoter. Treatment with HR73 (1 μM in DMSO) or DMSO was performed for 36 h after overnight infection. The average (± SEM) of four experiments is shown.

The identification of SIRT1 as an enzyme that catalyzes an important step in HIV transcription suggests that it could be targeted therapeutically. Splitomicin was identified as a small molecule inhibitor of the S. cerevisiae Sir2p protein [41]. While splitomicin did not inhibit human SIRT1, we identified a splitomicin derivative, called HR73, which is structurally related to a previously described inhibitor of Hst1, a homolog of Sir2p in yeast [42]. HR73 effectively inhibited the histone deacetylase activity of SIRT1 in vitro with an IC_50_ (concentration causing 50% inhibition) of less than 5 μM (Figure 6A and 6B). Treatment of HeLa cells with HR73 suppressed Tat-dependent HIV transcription in a dose-dependent manner (3-fold at approximately 1 μM) after transfection of the Tat vector and the HIV LTR luciferase construct (HIV LTR in Figure 6C). In separate experiments, HR73 induced hyperacetylation of another target of SIRT1, the tumor suppressor p53, at the same concentration (1 μM) (Wei Gu and EV, unpublished data). Importantly, HR73 (1 μM) did not suppress the activity of the RSV LTR, the promoter driving Tat expression in our experiments (RSV LTR in Figure 6C).

To examine the effect of HR73 on HIV infection, we generated infectious HIV particles using a molecular clone of HIV_NL4–3_ that contained the green fluorescent protein (GFP) open reading frame in place of the viral *nef* gene (HIV_NL4–3_ is a specific viral isolate) [43]. To restrict analysis to a single infection cycle, this clone also contained a frameshift mutation in the viral *env* gene. Viral particles were produced by cotransfection with a construct expressing the glycoprotein of the vesicular stomatitis virus (VSV-G). Jurkat T cells were incubated with viral supernatant for at least 18 h, washed to remove extracellular virus, and treated with HR73 (1 μM) or dimethyl sulfoxide (DMSO), as a control. We observed that HIV gene expression was reduced 5-fold in cells treated with HR73 as measured by GFP expression (HIV GFP in Figure 6D). In contrast, GFP expression in cells infected with an HIV-based lentiviral vector expressing GFP from the elongation factor 1α (EF-1α) promoter was not affected by HR73 treatment (HIV [EF-1α] in Figure 6D). These data confirm the selectivity of HR73 for HIV transcription and demonstrate that other steps in the viral life cycle, including reverse transcription, nuclear import, and integration, remain unaffected by HR73. These experiments collectively show that the SIRT1 deacetylase activity is required for HIV gene expression and establish SIRT1 as a potential drug target in the treatment of HIV-1 infection.

## Discussion

Our previous work documented that Tat acetylation by p300 at lysine 50 is a necessary step in Tat-mediated transactivation and leads to the recruitment of PCAF to the elongating polymerase. We now report the identification of SIRT1, an NAD^+^-dependent class III protein deacetylase, as a Tat deacetylase and regulator of Tat activity. Tat and SIRT1 coimmunoprecipitate and synergize to activate the HIV promoter. Conversely, knockdown of SIRT1 via siRNA inhibits Tat-mediated transactivation of the HIV LTR. Tat transactivation is defective in SIRT1-null MEFs and can be rescued by expression of SIRT1.

Recent observations indicate that SIRT1 can regulate transcription via histone-dependent and -independent mechanisms [20]. Since SIRT1 itself does not bind to DNA directly, targeted deacetylation of histones is thought to occur through its interaction with specific DNA binding factors, such as CTIP2 (chicken ovalbumin upstream promoter-transcription factor-interacting protein 2) [44], members of the hairy-related basic helix-loop-helix repressors (HES1 [hairy and Enhancer-of-split 1] and HEY2 [hairy and Enhancer-of-split related with YRPW motif 2]) [45], the MyoD/PCAF complex [25], and the N-CoR (nuclear receptor co-repressor) and SMRT (silencing mediator of retinoid and thyroid hormone receptor) co-repressors [46]. In addition, SIRT1 binds and deacetylates histone H1 and promotes the formation of facultative heterochromatin [33].

SIRT1 also deacetylates nonhistone factors, including the tumor suppressor p53 [21,22,23]. Detailed studies of acetylation of p53 and its regulation by SIRT1 have revealed a number of intriguing parallels to Tat regulation. p53 is acetylated by p300 at multiple lysine residues, including lysine 382, leading to an increased transcriptional activity of p53 [47]. Activation of p53 up-regulates the expression of genes whose products promote cell-cycle exit or apoptosis [22]. Therefore, p300 is a positive regulator of p53 activity, while SIRT1 negatively regulates p53 functions. SIRT1 deacetylation of p53 suppresses the induction of apoptosis and promotes cell survival in response to DNA damage [21,22]. Knockout mice for Sir2α show increased p53 acetylation after DNA damage leading to increased thymocyte apoptosis after ionizing radiation [48]. Interestingly, both SIRT1 and p53, and the Tat cofactor cyclinT1, are localized in promyelocytic leukemia protein bodies [23,49]. These unique nuclear substructures represent the natural accumulation sites of promyelocytic leukemia protein and are altered or disrupted in certain tumors and in response to different cellular stresses, including Tat expression (EV, unpublished data).

Interestingly, HDAC1 (a class I HDAC) has also been reported to deacetylate p53 [50], but the relative contributions of both HDAC1 and SIRT1 to p53 deacetylation remain unclear. In a previous study, an enhancement of Tat-mediated transactivation was observed in response to TSA, an inhibitor of class I and II HDACs, and was interpreted as evidence that Tat acetylation is under the control of a class I or II HDAC [7]. However, a positive effect of TSA on Tat-mediated transactivation can occur for a number of reasons that are independent of Tat acetylation levels. First, the HIV promoter can be activated by TSA in the absence of Tat, a phenomenon likely to be mediated by transcription factors responsive to TSA that bind to the HIV LTR, such as Sp1 (stimulatory protein 1), YY1 (Yin Yang 1), Eed (embryonic ectoderm development protein), and NF-κB/p65 [51,52,53]. Tat-independent activation of the HIV LTR by TSA has been documented both in vitro using chromatinized templates, and in vivo using cell lines containing integrated HIV genomes defective for Tat-mediated transactivation [54,55,56]. Second, TSA activates many promoters that are used to drive Tat expression in transient transfection experiments, making interpretations of these experiments problematic.

We have observed that Tat acetylation at lysine 50 is not specifically enhanced in response to TSA [11]. These results are supported by functional experiments with Tat mutants. In cotransfection experiments, enhanced transcriptional activity of Tat in response to TSA is preserved when lysine 50 is replaced by an alanine [7] or an arginine (MO, unpublished data). Although these experiments are complicated by the significant increase in Tat expression (due to the stimulatory role of TSA on Tat expression), they provide evidence that lysine 50 in fact might not be a target for a TSA-sensitive enzyme in HIV transcription.

Another system that offers intriguing parallels to our observation is the role of the SIRT1/MyoD/PCAF complex in myogenesis [25]. In this complex, PCAF acetylates both itself and MyoD. SIRT1, in contrast, deacetylates both PCAF and MyoD, leading to a retardation of myogenesis. The negative effect of SIRT1 in the regulation of muscle gene expression plays at two distinct levels. First, SIRT1 induces deacetylation of histones in the promoter regions. Second, SIRT1 deacetylates MyoD, which leads to an inhibition of its transcriptional activity [57]. Our previous experiments documented the specific interaction of acetylated Tat and PCAF via the PCAF bromodomain and the ARM region of Tat and the role of this interaction in Tat transactivation [13]. The possibility that Tat bound to the elongating polymerase and to PCAF is regulated by a PCAF-SIRT1 complex is intriguing and will be further explored.

In most identified sites of action, SIRT1 showed a negative activity on transcriptional regulatory mechanisms, resulting in an inhibition of target gene expression. In contrast, we have identified a positive regulatory activity of SIRT1 on Tat-mediated transactivation. According to our working model, Tat becomes acetylated at the level of the HIV promoter after binding to TAR. The acetylation of the Tat ARM can potentially affect other biological activities associated with this domain, including RNA binding, nuclear or nucleolar import, and protein stability. Tat acetylation could also be involved in the delivery of Tat to the transcription complex, and/or might moderate interactions with cyclinT1/CDK9 prior to transcription. However, we provided evidence that Tat acetylation leads to a dissociation of Tat from cyclinT1 and TAR and to its transfer to the elongating polymerase [11]. Acetylated Tat, bound to the elongating polymerase, recruits the transcriptional coactivator PCAF via its acetyl group and the bromodomain of PCAF [12,13].

An unresolved question raised by this model is the fate of acetylated Tat at the end of the transcription cycle. Since acetylated Tat cannot recruit cyclinT1 to the TAR element, a new transcription cycle would require either the synthesis of a new unacetylated Tat protein or the deacetylation of Tat. Tat concentrations are limiting early in the virus life cycle since Tat expression is under the control of the basal LTR. Tat recycling is therefore likely to represent a rate-limiting step in HIV transcription early during the replicative cycle of the virus. Experiments presented in this manuscript demonstrate that SIRT1 catalyzes this rate-limiting step in HIV transcription.

These observations raise a number of additional questions. The demonstration that Tat transactivating activity is controlled by SIRT1 connects HIV transcription with the metabolic state of the cell. The HDAC activity of SIRT1 requires NAD^+^ as a cofactor. NAD^+^ and its reduced form NADH serve as cofactors in many metabolic and stress reactions involving oxidation and reduction. Changes in the cellular NAD^+^/NADH ratio may regulate SIRT1 enzymatic activity, Tat transcriptional activity, and HIV replication and pathogenesis.

The site of Tat deacetylation is unknown at this point. We hypothesize that the deacetylation could be triggered by molecular events linked with the end of the transcriptional elongation process. Deacetylation of Tat by SIRT1 could lead to its dissociation from the elongating polymerase and from PCAF. The previous demonstration that PCAF and SIRT1 can be coimmunoprecipitated from cells supports this model [25].

The identification of SIRT1 as an enzyme that catalyzes a rate-limiting step in HIV transcription suggests that it could be targeted therapeutically. Since our preliminary experiments indicated that splitomicin, an inhibitor of the S. cerevisiae Sir2 enzymatic activity, did not inhibit the mammalian sirtuins, we screened several compounds structurally related to splitomicin for their ability to inhibit the mammalian sirtuins. This process led to the identification of HR73, an inhibitor that suppresses SIRT1 enzymatic activity in vitro at low micromolar concentrations. This inhibitor induced p53 hyperacetylation in vivo (unpublished data) and significantly decreased HIV transcription. These results validate SIRT1 as a novel therapeutic target for HIV infection. Future efforts will analyze in additional molecular detail the role of Tat acetylation and deacetylation in HIV transcription and replication.

## Materials and Methods

### 

#### Cells and plasmids

HeLa, HEK 293, and Jurkat cells (obtained from the American Type Culture Collection, Manassas, Virginia, United States), and wild-type or Sir2α knockout MEFs [37] were maintained under standard cell culture conditions. The HIV LTR luciferase plasmid [58], the RSV LTR luciferase construct [11], the 5xUAS luciferase construct [59], the LTR_ΔNF-κB_-luciferase, the full-length (101 amino acid) CMV-Tat/FLAG and CMV-Tat/T7 expression vectors [8], the full-length (101 amino acid) RSV-Tat expression vector [60], the full-length cyclinT1 expression vector [5], the Gal4-VP16 expression vector [13], the human SIRT1–7 expression vectors with a C-terminal FLAG tag [30], as well as wild-type and mutant human SIRT1 and SIRT1H363Y containing a C-terminal MYC (myelocytomatosis oncogene) tag [23] were previously described.

The SIRT1 cDNA was subcloned to generate a C-terminal HA-tagged fusion in a derivative pcDNA 3.1 (+) backbone (HA vector) by standard PCR-based strategies. The mutant CMV-Tat/FLAG expression vector TatK50R was generated by site-directed mutagenesis with the following primers. Forward, 5′- CCTATGGCAGGAGGAAGCGGAGACAGCG-3′ and reverse, 5′- CGCTGTCTCCGCTTCCTCCTGCCATAGG-3′. The mutant HIV LTR luciferase constructs (nucleotides 1–791) were generated by site-directed mutagenesis with the following primers. TAR Δbulge (T_223_→A) forward, 5′- GGTTAGACCAGAACTGAGCCTGGGAGC-3′ and reverse, 5′- GCTCCCAGGCTCAGTTCTGGTCTAACC-3′. The TAR Δloop mutation (C_230_→A, T_231_→G, and G_234_→T) was generated with the following primers. Forward, 5′- GGTTAGACCAGATCTGAGCAGGGTAGCTCTCTGGCTAACTAGGG-3′ and reverse, 5′- CCCTAGTTAGCCAGAGAGCTACCCTGCTCAGATCTGGTCTAACC-3′.

The CMV-luciferase construct was generated by cloning the firefly luciferase gene as a HindIII/BamHI fragment obtained from pGL2 Basic (Promega, Madison, Wisconsin, United States) into pcDNA3.1 (Invitrogen, Carlsbad, California, United States). The CMV-GFP expression plasmid is commercially available (Clontech, Palo Alto, California, United States).

#### Synthesis of Tat and HR73

Synthesis of 72-amino acid Tat proteins was as described [11,13]. HR73 was synthesized starting from Phenylmeldrum's acid and 6-bromo-1-dimethylaminomethyl-2-naphthol as described [61]. Identity and purity were assured by mass, infrared, and NMR spectroscopy as well as by elemental analysis.

#### In vitro Tat deacetylation assay

Human SIRT1–7 FLAG-tagged plasmids were transfected in HEK 293 cells with Lipofectamine reagent (Invitrogen). Cells were lysed 24 h after transfection with lysis buffer (50 mM Tris-HCl [pH 7.5], 0.5 mM EDTA, 0.5% NP40, and 150 mM NaCl) in the presence of protease inhibitors (Roche Molecular Biochemicals, Indianapolis, Indiana, United States). Equal amounts of total proteins were immunoprecipitated with α-FLAG M2 agarose (Sigma, St Louis, Missouri, United States), for 2 h at 4 °C. Immunoprecipitated material was washed twice with IP buffer and one time with SIRT deacetylation buffer (50 mM Tris-HCl [pH 9], 4 mM MgCl_2_, and 0.2 mM DTT). The beads were resuspended in 100 μl of SIRT deacetylation buffer containing 1 μg synthetic Tat (72 amino acids) carrying an N-terminal biotin label and an acetyl group at position 50 [11]. Reactions containing TSA (400 nM; WACO Bioproducts, Richmond, Virginia, United States) or nicotinamide (5 mM; Sigma) were preincubated for 10 min at room temperature. After addition of NAD^+^ (1 mM), reactions were incubated for 2 h at room temperature with constant agitation. Reactions were stopped by the addition of SDS loading buffer, boiled, and after brief centrifugation, analyzed by WB with rabbit α-AcTat antibodies [11] or SA-HRP (Jackson Immunoresearch Laboratories, West Grove, Pennsylvania, United States). SIRT1–7 proteins were detected with polyclonal α-FLAG antibodies (Sigma).

The histone deacetylation assay with recombinant SIRT1 (1–1.3 U/reaction; Biomol, Plymouth Meeting, Pennsylvania, United States) was performed as described previously for SIRT2 [30] in 100 μl of SIRT deacetylase buffer containing NAD^+^ (Sigma) and enzymatically [^3^H]-acetylated histone H3 peptide (amino acids 1–24) [62]. Splitomicin (a gift from Julian Simon, Fred Hutchinson Cancer Research Center, Seattle, Washington, United States) and HR73 in DMSO were added to the reactions at the indicated concentrations with all components of the reactions in the absence of NAD^+^ for 10 min at room temperature prior to the initiation of the reaction by addition of NAD^+^ (1 mM).

#### Coimmunoprecipitation experiments

HEK 293 cells were cotransfected in duplicate with expression vectors for CMV-Tat/FLAG; CMV-Tat/T7 or CMV-TatK50R/FLAG; and the SIRT1/HA or SIRT1-, SIRT2-, and SIRT6-FLAG expression vectors or the respective empty vector controls using Lipofectamine reagent (Invitrogen). Cells were lysed after 24 h in 250 mM NaCl, 0.1% NP40, 20 mM NaH_2_PO_4_ (pH 7.5), 5 mM EDTA, 30 mM sodium pyrophosphate, 10 mM NaF, and protease inhibitors (Roche Molecular Biochemicals). Duplicates were pooled, and 1 mg of lysate was immunoprecipitated either with monoclonal α-HA (Roche Molecular Biochemicals) together with protein G-Sepharose (Amersham Biosciences, Piscataway, New Jersey, United States) with α-FLAG M2 agarose (Sigma) or α-T7-agarose (Amersham Biosciences) for 2 h at 4 °C. Beads were washed three times in lysis buffer, boiled in SDS loading buffer, and analyzed by WB with polyclonal α-FLAG (Sigma), monoclonal α-HA (Roche), or monoclonal α-T7 (Novagen, Madison, Wisconsin, United States) antibodies. For the IP of Tat with endogenous SIRT1, HEK 293 cells were transfected only with CMV-Tat/FLAG or the CMV-empty vector using Lipofectamine reagent. Cell lysates were immunoprecipitated with rabbit α-SIRT1 antibodies (generated against amino acids 506–747) together with protein G-Sepharose (Amersham Biosciences). Immunoprecipitated material was analyzed by WB with the M2 α-FLAG antibody (Sigma) or rabbit α-SIRT1 antibodies.

For in vitro interactions, 10 U of recombinant SIRT1 (Biomol) was incubated with biotinylated synthetic Tat or acetylated Tat proteins (0, 0.25, 1, and 4 μg) together with streptavidin-Sepharose (Amersham Biosciences) in lysis buffer in the presence of 5 mM nicotinamide (Sigma) for 3 h at 4 °C. Pelleted beads were washed three times in lysis buffer, resuspended in SDS loading buffer, and analyzed by WB with polyclonal α-SIRT1 antibodies, rabbit α-AcARM, or SA-HRP (Jackson Immunoresearch Laboratories).

#### RNAi and transfection experiments

Double-stranded siRNAs directed against nucleotides 408–428 in the SIRT1 mRNA or control GL3 siRNAs (both Dharmacon Research, Lafayette, Colorado, United States) were transfected into HeLa cells plated in six-well plates with Oligofectamine reagent according to the manufacturer's guidelines (Invitrogen). The mutant SIRT1 siRNA was identical to SIRT1 siRNA except for a two-nucleotide mismatch between the target mRNA for SIRT1 and the antisense strand of siRNA at nucleotides 418 and 419. After 48 h, cells were retransfected with the HIV LTR luciferase construct (200 ng) together with increasing amounts of CMV-Tat expression vectors (0, 50, 100, 200, 400, and 800 ng in GL3/SIRT1 siRNA experiments; 0, 2, 20, and 200 ng in SIRT1/mutant SIRT1 siRNA experiments) and corresponding amounts of empty pcDNA3.1 vector (Invitrogen). In the control experiment, CMV-Tat was replaced by the CMV-luciferase construct, and HIV LTR luciferase was replaced by an HIV LTR promoter construct driving the expression of chloramphenicol acetyl transferase (CAT) [55]). Cells were harvested 24 h later and either processed for luciferase assays (Promega) or WB of total cell extracts with polyclonal α-SIRT1 or α-actin (MP Biochemicals, Aurora, Ohio, United States) antibodies.

In cotransfection experiments, human CMV-SIRT1 or CMV-SIRT1H363Y (600 ng) was cotransfected into HeLa cells plated in six-well plates with the HIV LTR luciferase reporter (200 ng) or the LTR_ΔNF-κB_-luciferase reporter (200 ng) and increasing amounts of RSV-Tat (0, 2, 20, and 200 ng) using the Lipofectamine reagent (Invitrogen). In the control experiment, RSV-Tat was replaced by RSV-luciferase (200 ng), and the HIV LTR luciferase construct was replaced by the HIV LTR CAT reporter. In transfections with HR73, HeLa cells were cotransfected with the HIV LTR luciferase reporter (200 ng) and RSV-Tat expression vectors (0, 20, and 200 ng) or the empty vector using Lipofectamine reagent. The RSV-luciferase construct was used as described above. After 4 h incubation with the DNA/Lipofectamine mix, the culture medium was changed and supplemented with indicated concentrations of HR73 dissolved in DMSO or DMSO alone. Cells were harvested 8 h later and processed for luciferase assays.

#### Microinjection experiments

Subconfluent MEFs (70%) were grown on Cellocate coverslips (Eppendorf, Westbury, New York, United States), and nuclear microinjections were performed at room temperature with an automated injection system (Eppendorf Micromanipulator 5171 together with Eppendorf Transjector 5246). Samples were prepared as a 20 μl injection mix containing the HIV LTR luciferase reporter or 5xUAS luciferase (each 100 ng/μl), RSV-Tat (10 ng/μl) or Gal4-VP16 (50 ng/μl), CMV-cyclinT1 (100 ng/μl), CMV-SIRT1 (100 or 300 ng/μl), together with CMV-GFP (50 ng/μl) in sterile water. At 6 h after microinjection, cells were examined under a Nikon Eclipse TE300 inverted fluorescent microscope (Nikon, Tokyo, Japan) to determine the number of GFP-positive cells, washed in cold phosphate buffer, and stored at −70 °C for luciferase assays (Promega). In HeLa cells, synthetic Tat or AcTat proteins (each 30 or 100 ng/μl) were coinjected with the wild-type or mutant HIV LTR luciferase reporters (each 100 ng/μl) together with CMV-GFP (50 ng/μl), and harvested 4 h after injection. Cells were treated immediately after injection with DRB (10 or 50 μM; Sigma), TSA (400 nM), or nicotinamide (5 mM). Microinjections in siRNA-treated cells were performed 48 h after siRNA transfection.

#### Viral infection experiments

The HIV molecular clone HIV-R7/E^−^/GFP containing the GFP open reading frame in place of the *nef* gene and a frameshift mutation in the *env* gene, as well as the method to generate pseudotyped viral particles with VSV-G, were previously described [43]. The number of infective particles per milliliter was established by infecting 3 × 10^5^ Jurkat cells with different amounts of viral suspension. The titer of the viral stock was measured by flow cytometric analysis of GFP expression 48 h after infection. The pHR′-EF-1α/GFP construct is a minimal nonreplicative HIV-1 genome containing a heterologous promoter, EF-1α, driving GFP expression [63]. Viral particles were produced by cotransfection of the VSV-G-encoding pMD.G and the HIV-based packaging vector pCMVΔR8.91 as described [64]. All vectors for the production of HIV-based lentiviral vectors were provided by Didier Trono, University of Geneva, Switzerland. Jurkat T cells were incubated overnight with HIV-R7/E^−^/GFP or pHR′-EF-1α/GFP viral particles at a theoretical multiplicity of infection of 0.5 in 24-well plates. Cells were repeatedly washed and resuspended in fresh medium containing HR73 (1 μM) or DMSO alone. Viral infection was monitored 36 h later by flow cytometry analysis using a Calibur FACScan (Becton Dickinson, Palo Alto, California, United States).

## Abbreviations

AcTat: synthetic Tat protein carrying an acetylated lysine at position 50
ARM: arginine-rich motif
CAT: chloramphenicol acetyl transferase
CDK9: cyclin-dependent kinase 9
CMV: cytomegalovirus
DMSO: dimethyl sulfoxide
DRB: 5,6-dichlorobenzimidazole riboside
EF-1α: elongation factor 1α
GFP: green fluorescent protein
HA: influenza hemagglutinin
HDAC: histone deacetylase
HIV: human immunodeficiency virus
IP: immunoprecipitation
LTR: long terminal repeat
MEF: mouse embryonic fibroblast
MyoD: myoblast determination protein
NAD^+^: nicotinamide adenine dinucleotide
NF-κB: nuclear factor kappa B
PCAF: p300/CREB-binding protein-associated factor
RSV: Rous sarcoma virus
SA-HRP: streptavidin-horseradish peroxidase conjugate
SEM: standard error of the mean
Sir2p: silent information regulator 2 protein
SIRT: sirtuin
siRNA: small interfering RNA
TAR: *trans*-acting responsive element
TSA: trichostatin A
UAS: upstream activating sequence
VSV-G: glycoprotein of the vesicular stomatitis virus
WB: Western blot
